# Epigenetic Immune Cell Counting to Analyze Potential Biomarkers in Preterm Infants: A Proof of Principle in Necrotizing Enterocolitis

**DOI:** 10.3390/ijms24032372

**Published:** 2023-01-25

**Authors:** Michiel H. D. Schoenaker, Mara O. Zuiderwijk, Vincent Bekker, Robbert G. M. Bredius, Jeannette Werner, Janika J. Schulze, Mirjam van der Burg, Maartje Blom

**Affiliations:** 1 Willem Alexander Children’s Hospital, Laboratory for Pediatric Immunology, Department of Pediatrics, Leiden University Medical Center, 2300 RC Leiden, The Netherlands; 2 Willem Alexander Children’s Hospital, Division of Neonatology, Department of Pediatrics, Leiden University Medical Center, 2300 RC Leiden, The Netherlands; 3 Department of Pediatrics, Willem Alexander Children’s Hospital, Leiden University Medical Center, 2300 RC Leiden, The Netherlands; 4 Department of Research and Development, Epimune GmbH, 12489 Berlin, Germany

**Keywords:** epigenetic immune cell counting, regulatory T cell, Th17 cell, necrotizing enterocolitis, neonate, preterm

## Abstract

Epigenetic immune cell counting is a DNA (de)methylation-based technique which can be used to quantify lymphocyte subsets on dried blood spots (DBS). The foregoing techniques allow for a retrospective investigation of immune cell profiles in newborns. In this study, we used this technique for determining lymphocyte subcounts as a potential biomarker for necrotizing enterocolitis (NEC). We investigated whether this technique can be implemented in the field of neonatology, by testing whether regulatory T cell (Treg) levels are pre-existently low in preterms with NEC. Newborn screening (NBS) cards from 32 preterms with NEC and 32 age- and weight-matched preterm controls, and 60 healthy term newborns, were analyzed. Relative and absolute cell counts were determined for CD3+, CD4+, CD8+, Th17, and Treg T cells. For both relative and absolute cell counts of CD3+, CD4+, CD8+, and Th17 T cells, significant differences were found between healthy term controls and both preterm groups, but not between preterm groups. For Tregs, no significant differences were found in either relative or absolute counts between any of the newborn groups. This study demonstrates the principle of epigenetic immune cell counting to analyze lymphocyte subsets in preterm neonates.

## 1. Introduction

Epigenetic immune cell counting is an innovative technique which gives the opportunity to quantify various lymphocyte subsets in a single drop of blood [1]. It is a DNA (de)methylation-based technique that allows for quantitative assessment of immune cells [2]. As DNA is a stable substrate, epigenetic qPCR can even be performed on dried blood spots (DBS) such as newborn screening (NBS) cards. The technique has previously been used in the field of oncology, in both research and clinical settings [1]. This unique approach provides the opportunity to retrospectively investigate lymphocyte profiles in preterm newborns on a Neonatal Intensive Care Unit (NICU) without additional blood withdrawal in this fragile patient group.

Necrotizing enterocolitis (NEC) is a common disease among preterm newborns and is associated with high mortality and morbidity, including failure to thrive, complications of the gastro-intestinal tract, and an impaired developmental outcome [3]. Treatment of NEC consists of antibiotics, and, in the case of bowel perforation, acute surgery [4]. Although this disease has been known for a long time, the pathophysiology is not clarified yet, and is probably multifactorial. One of the main thoughts is that acute inflammation leads to bowel ischemia and necrosis [5]. An important pathophysiologic hypothesis mentions the disbalance in the developing immune system of preterm newborns, with low numbers of regulatory T cells (Tregs) in preterm newborns with a NEC, and therefore show a lack or dysfunction of anti-inflammatory effects [6]. Both mice and human studies have shown low numbers of T-regs during NEC in both peripheral blood and bowel sections [7,8]. A lack of Tregs could contribute to a more pro-inflammatory environment which could be the cause of acute inflammation of the bowel and the onset of NEC. In our recent scoping review, we discussed several pathophysiological pathways in which the (lack of) functioning Tregs could play a role in preventing the onset of NEC [9]. The question remains whether the number of Tregs is pre-existently decreased in preterm neonates ultimately developing a NEC or whether this observation is a bystander effect. 

Epigenetic immune cell counting provides the opportunity to analyze and determine relative numbers of various lymphocyte subsets in original newborn screening (NBS) cards from NEC patients. This study shows how the technique can be implemented in the field of neonatology by testing whether potential pre-existent low Treg numbers occur in preterms with NEC as a proof of principle. 

## 2. Results

### 2.1. Principle of Epigenetic Immune Cell Counting

**Figure 1:**
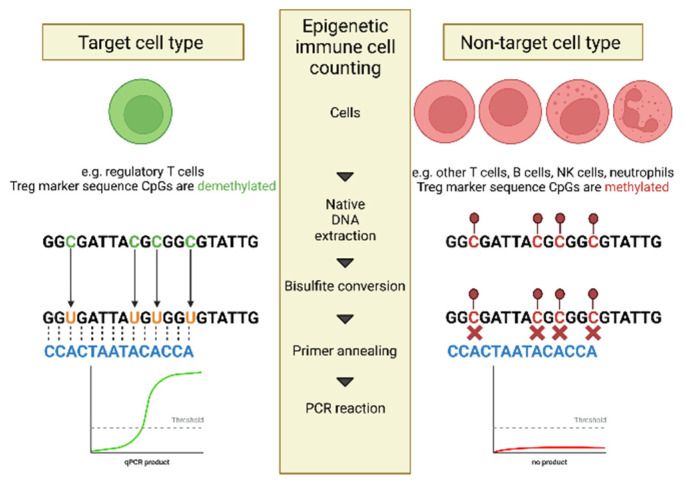
The principle of epigenetic immune cell counting by amplification of cell type-specific demethylated genomic regions, adapted from Epimune GmbH.

Epigenetic immune cell counting is a DNA (de)methylation-based, quantitative assessment of immune cells. Genomic DNA is treated with ammonium bisulfite which results in the conversion of unmethylated CpG dinucleotides to TpGs. Methylated CpGs remain unaltered, therefore epigenetic marks are translated into sequence information. Un-methylated specific primers and probes allow for discrimination of both variants, and only cell-type specific un-methylated regions are amplified (Figure 1). DNA is a stable substrate preserving epigenetic modifications, which means that epigenetic qPCR is resistant to loss of cell integrity and can be performed on fresh or frozen blood, or dried blood spots [10]. 

### 2.2. Relative Lymphocyte Counts in Preterms with NEC

Epigenetic immune cell counting was used to measure the relative counts of CD3+, CD4+, CD8+, Treg, and Th17 T cell counts from DBS of 60 healthy control term newborns, 32 preterm newborns with NEC, and 32 matched preterm controls. Counts of the cell-type specific genomic regions were related to GAPDH counts, which was used as a universal denominator since it is invariably unmethylated [11].

**Figure 2:**
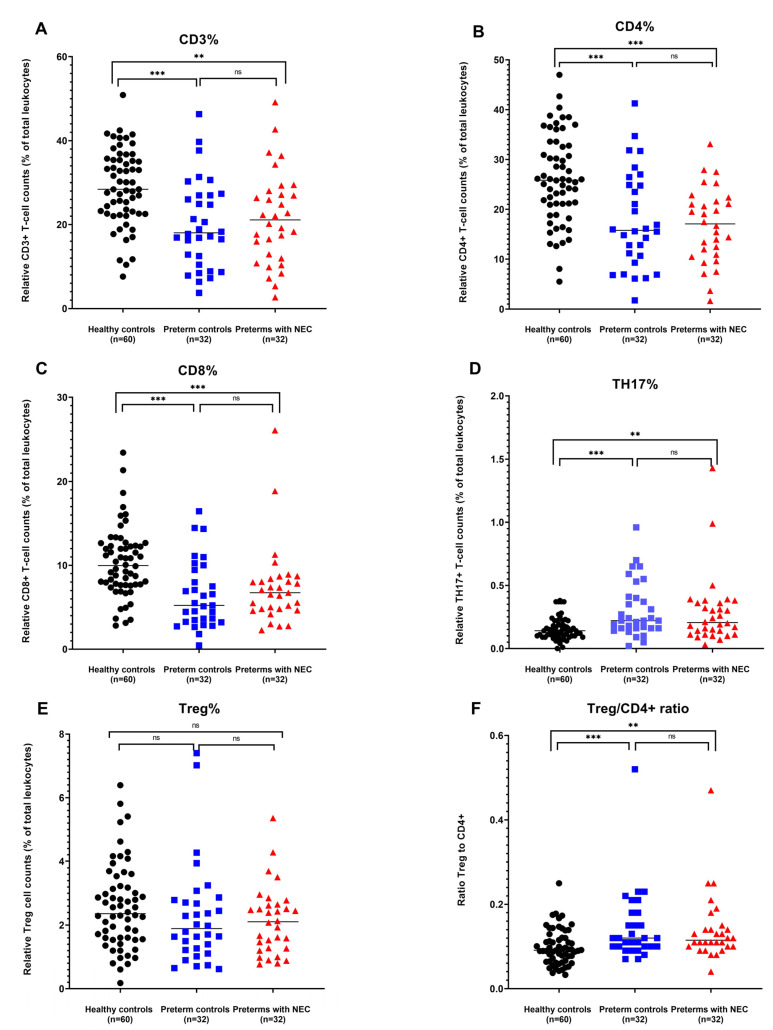
(**A**–**E**) Relative (epi) CD3+, CD4+, CD8+, Th17, and Treg T cell counts (% of demethylated gene copies of total GAPDH demethylated copies) of healthy term controls (*n* = 60), preterm controls (*n* = 32) and preterms with NEC (*n* = 32), presented on the X-axis. Y-axis displays the relative cell count as percentage of total leukocytes. (**F**) Relative Treg/CD4 ratio. Groups are presented on the X-axis, with the Treg/CD4+ ratio on the Y-axis. Pairwise significances are represented at the top of the graph, with ns meaning non-significant, ** meaning *p* < 0.01, *** meaning *p* < 0.001. Black lines indicate the median value.

We observed that there were significantly higher relative CD3+ T cell counts in healthy term newborn controls compared to preterm controls and preterms with NEC (preterm control (PC): *p* < 0.001; preterms with NEC (PN): *p* = 0.001), which is in accordance with previous studies [12]. However, no significant differences were observed in relative CD3+ T cell counts between preterm controls and preterms with NEC (*p* = 0.577) (Figure 2A).

The same trend was observed for relative counts for CD4+ and CD8+ T cells. No significant differences were observed when comparing relative CD4+ and CD8+ T cell counts between preterm controls and preterms with NEC (*p* = 0.889; and *p* = 0.257, respectively). When comparing both groups of preterms to healthy newborn controls, all preterms have significantly lower relative CD4+ (PC: *p* < 0.001; PN: *p* = 0.000) and CD8+ T cell counts (PC: *p* < 0.001; PN: *p* = 0.000) (Figure 2B,C).

In previous studies investigating NEC, it has been shown that levels of Tregs were decreased, whereas levels of Th17 cells were increased in lamina propria of mice after NEC induction [13]. Additionally, studies investigating cord blood showed higher frequencies of Th17 cells in preterms compared to term human newborns [14]. Because of these findings, we investigated both the relative counts of Tregs and Th17 cells between preterms and healthy term controls.

Our results showed no significant differences in relative Th17 cell counts between preterms with NEC and preterm controls (*p* = 0.436), but both sets of preterms did show higher relative Th17 cell counts compared to healthy controls in DBS (PC: *p* < 0.001; PN: *p* = 0.003) (Figure 2D).

To determine relative levels of Tregs, the FOXP3 gene was used as Treg specific genomic region, as this gene has been shown to be a transcription factor which induces Treg differentiation [15]. No significant differences were observed in the relative Treg cell counts between healthy newborn controls and preterm controls, healthy newborn controls and preterms with NEC, or preterm controls and preterms with NEC (*p* = 0.14; *p* = 0.124; and *p* = 0.867, respectively) (Figure 2E). Further studies in surgically resected ileum from preterms with NEC showed that these patients had a lower Treg to CD4+ T cell ratio compared to the ileum of preterms without NEC [6]. Therefore, the Treg/CD4+ ratio was determined for this study population. No significant differences were observed in the Treg/CD4+ ratio between preterms with NEC and preterm controls (*p* = 0.917). The Treg/CD4+ ratio was significantly increased between preterm groups and the healthy newborn controls (PC: *p* < 0.001; PN *p* = 0.001) (Figure 2F).

### 2.3. Absolute Lymphocyte Counts in Preterms with NEC

The exact amount of blood in a dried blood spot is unclear, but it is roughly estimated to be 3.0 µL per 3.2 mm punch. With this estimation, absolute cell counts were calculated. A plasmid of known copy number was spiked to the sample and quantified with specific primers and probes. The ratio measured to initially applied copies of spike plasmid allows for the inference of an initial number of GAPDH copies, and thus cells in the sample. The absolute cell counts were determined for CD3+, CD4+, CD8+, Th17, and Treg T cells. To determine Treg cell counts, FOXP3 was once again used. In this analysis, 28 preterms with NEC and 28 age- and weight-matched preterm controls were used, as the final four preterms in both groups were included after this analysis was performed.

**Figure 3:**
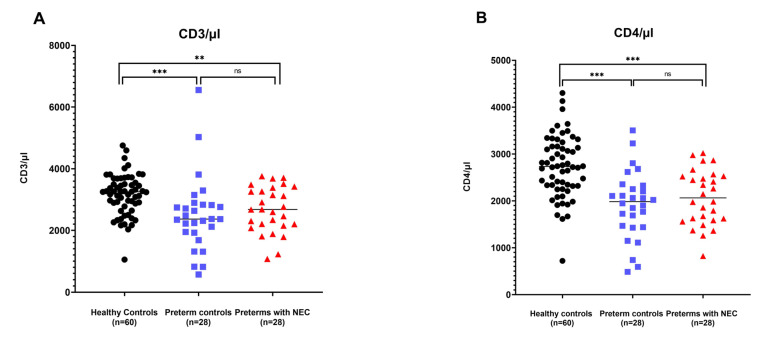

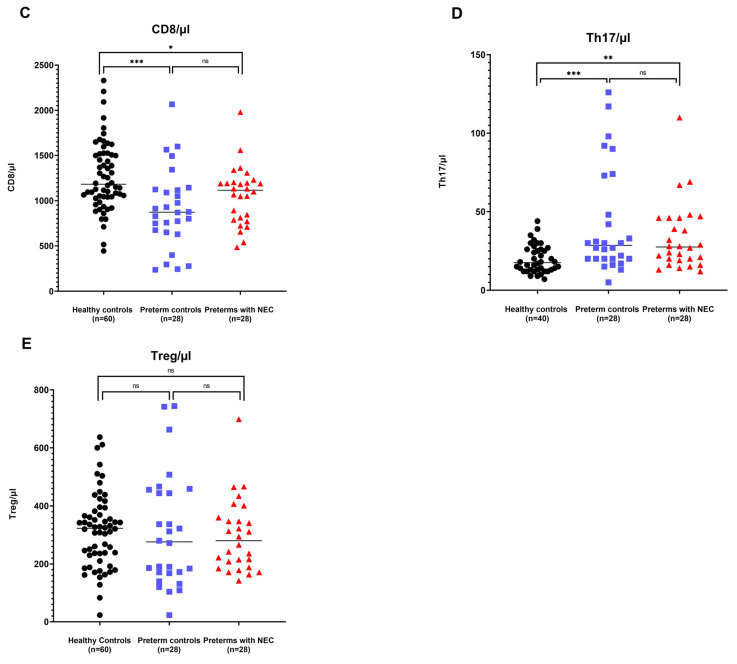
(**A**–**E**) Estimated absolute CD3+, CD4+, CD8+, Th17, and Treg T cell counts measured in dried blood spots of healthy term controls (*n* = 60), preterm controls (*n* = 28), and preterms with NEC (*n* = 28), presented on the X-axis. Y-axis depicts the number of cells per µL. Pairwise significances are represented at the top of the graph, with ns meaning non-significant, * meaning *p* < 0.05, ** meaning *p* < 0.01, *** meaning *p* < 0.001.

For absolute CD3+ T cell counts, no differences were observed between preterms with NEC and preterm controls (*p* = 0.278), but a number of CD3+ T cell counts were observed in healthy controls compared to preterms (PC: *p* < 0.001; PN: *p* = 0.006) (Figure 3A). 

The same trends can be seen for CD4+ T cells. Between healthy controls and preterm controls, and healthy controls and preterms with NEC, significant decreases in absolute CD4+ T cell counts were observed in the preterms (PC: *p* < 0.001; PN: *p* < 0.001). Once again, no differences in absolute CD4+ T cell counts were found between the two preterm groups (*p* = 0.3925) (Figure 3B).

Regarding the absolute CD8+ T cell counts, an increase was shown in healthy controls compared to preterm controls, as well as preterms with NEC (PC: *p* < 0.001; PN: *p* = 0.027). Between the preterm controls and preterms with NEC, no differences in absolute CD8+ T cell counts were observed (*p* = 0.086) (Figure 3C). 

In the analysis of absolute Th17 T cell counts, it was once again observed that their number significantly increased when comparing healthy controls to either of the preterm groups (PC: *p* < 0.001; PN: *p* = 0.001). No differences were seen between preterm controls and preterms with NEC (*p* = 0.5603) (Figure 3D).

For the absolute counts Tregs, no significant differences were observed between the heathy controls and either of the preterm groups (PC: *p* = 0.431; PN: *p* = 0.388), nor between the two preterm groups themselves (*p* = 0.705) (Figure 3E). 

### 2.4. Influence of Stage of NEC on Relative Treg Cell Counts

**Figure 4:**
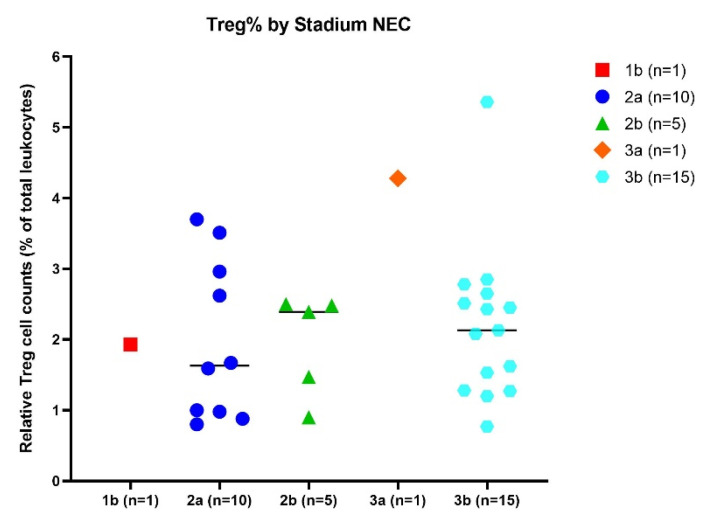
Different stages of NEC and relative Treg cell counts. The X-axis displays the NEC stage, while the Y-axis shows the relative Treg cell count. Black lines represent the median value per group.

Clinical presentation of NEC may vary among infants, specifically regarding progression, which can range from insidious and slow to progressive and rapid. Disease severity can be assigned based on staging criteria for NEC: 1b suspected NEC, 2a proven NEC (mildly ill; *n* = 1), 2b proven NEC (moderately ill; *n* = 10), 3a advanced NEC (severely ill, bowel intact; *n* = 1), and 3b advanced NEC (severely ill, bowel perforated; *n* = 15) [16]. It was hypothesized that lower numbers of Treg might be associated with higher stages of NEC. No significant differences in relative Treg cell numbers were shown between any of the different NEC stages (Figure 4). 

### 2.5. Change in Relative Number of Tregs over Time

**Figure 5:**
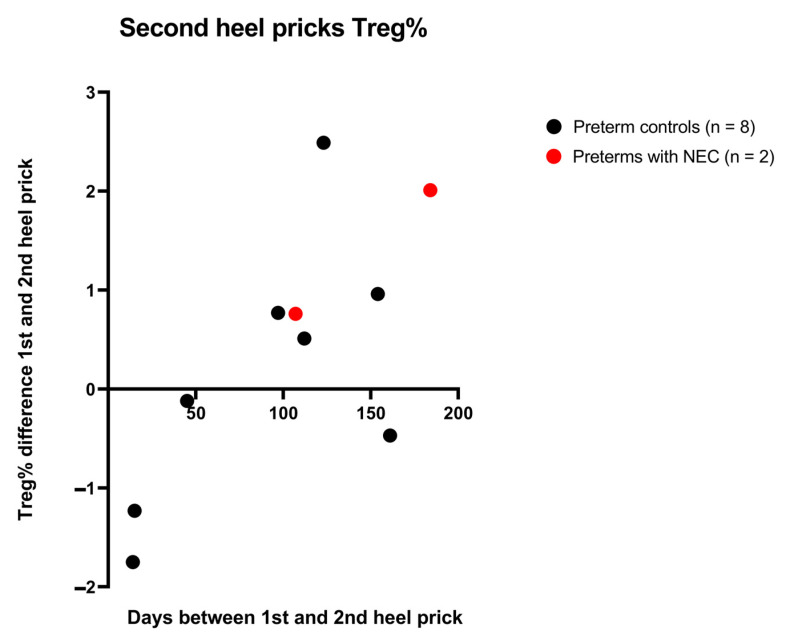
The number of days between the first and second heel prick (X-axis) and the difference in relative Treg cell counts between the heel pricks (Y-axis). Preterms with NEC are indicated in red (*n* = 2). Preterm controls are indicated in black (*n* = 8).

To confirm previous studies that indicate that lymphocyte subpopulations, including Tregs, increase with time as the immune system develops, epigenetic immune cell counting was performed in ten second heel prick samples that were available within the cohort. Second heel pricks are requested in the case of inconclusive results due to blood transfusions, prematurity or other factors. Two preterms with NEC and eight control preterms received a second heel prick. There was significant positive correlation between the days between the first and second heel prick and the relative Treg cell counts (Pearson r correlation 0.7228, *p* = 0.0182), indicating that the relative Treg cell count increased over time in individual preterm newborns (Figure 5).

## 3. Discussion

In this study, we successfully demonstrated the concept of epigenetic immune cell counting as a promising technique for lymphocyte quantification in a NICU cohort. Besides using this technique to quantify lymphocytes at a single time point, we have also shown that it can be used to determine changes in lymphocyte levels in an individual newborn over time. Epigenetic qPCR has proven to be a new valuable tool for immunological research and immunodiagnostics in preterm newborns. Additionally, we have shown that the number of relative FOXP3+ T-cell or Treg counts is not decreased in preterm newborns developing a NEC at the time of sample collection of the heel prick card (72 to 168 h after birth) compared to age- and weight-matched preterm controls. We did show that when comparing preterms with term controls, CD3+, CD4+, and CD8+ cell numbers decreased in the preterms, while Th17 cell counts increased. The changes in T cell counts are expected, as the immune system of preterm newborns is underdeveloped and in a different developmental stage compared to term newborns.

Epigenetic immune cell counting, especially in combination with an accessible cohort of heel prick cards, provides a rare opportunity to perform immunodiagnostics without additional blood withdrawal. Usually, cord blood is used to analyze preterm blood in research settings on a NICU. However, Olin et al. demonstrated, in a large longitudinal study, that cord blood samples are not representative for immune parameters in newborns [17]. Therefore, alternative study techniques such as epigenetic qPCR are required to prove hypotheses related to the developing immune system in neonates. 

With regards to our hypothesis on whether Tregs are low prior to NEC development, it seems that Tregs and other T cells, including CD4+ T cells, CD8+ T cells, and Th17+ cells in NBS cards, cannot be used as predictors or biomarkers for the occurrence of NEC in preterm newborns. Even though our study was limited by a small sample size and a single time point measure, it was strengthened by the ability to perform a comparison between NEC patients and age- and weight-matched comparable preterm controls. In most NICU research close matching is more difficult, resulting in heterogenous populations which may bias outcomes. Additionally, by using original NBS cards from the Dutch NBS program, no extra blood had to be withdrawn. 

The question of whether NEC is preceded by a reduction of Treg numbers remains to be answered, as this study showed measurements at a single timepoint and has limited data on second heel pricks collected at a later date. Future research should include prospective sample collection in a NICU cohort at multiple time points to determine whether the drop of Treg numbers during NEC is a bystander effect or part of a causal pathway in NEC development. Sample collection might be challenging due to the low body weight of the patient cohort, and ethically questioned, since newborns that will not develop NEC may be included. Blood drops for research could be collected simultaneously with regular checks to prevent additional withdrawal. For example, this procedure could be combined with heel prick checks for hyperbilirubinemia, as all preterms are at risk of this. 

This innovative technique creates an opportunity to perform serial immunodiagnostics to analyze trends of immune cells without the burden of extra blood withdrawal when used on NBS cards or blood spots taken at regular checks. From both a patient and a research perspective, the application of epigenetic immune cell counting is an advantage in the current research setting on the NICU.

## 4. Materials and Methods

### 4.1. Study Population

Preterm infants (gestational age <32 weeks) born between 2015 and 2020 at the Leiden University Medical Center (LUMC) were selected for inclusion. A control group with preterms was included from a NICU-database and matched on clinical parameters, in particular age and birth weight. Epigenetic immune cell counting was performed on original heel prick blood of anonymized healthy term controls (*n* = 60), preterms with NEC (*n* = 32), and matched control preterms (*n* = 32). Original NBS cards were retrieved from the archives of the National Institute for Public Health and the Environment (RIVM). Newborns with NEC were categorized according to modified Bell Staging criteria: 1b suspected NEC, 2a proven NEC (mildly ill; *n* = 1), 2b proven NEC (moderately ill; *n* = 10), 3a advanced NEC (severely ill, bowel intact; *n* = 1), and 3b advanced NEC (severely ill, bowel perforated; *n* = 15) [16]. This study was approved by the Medical Ethics Committee of Leiden-Den Haag-Delft (METC-LDD, reference B21.001). Samples of newborns whose parents objected to the use of dried blood spots for scientific research or used the opt-out option for research at the NICU were omitted from the study. The use of NBS cards was approved by the National Institute for Public Health and the Environment (RIVM).

### 4.2. Experimental Approach

Epigenetic immune cell counting was performed by amplification of cell-type-specific demethylated genomic regions according to the protocol of the manufacturer (Epimune GmbH, Berlin, Germany). DBS samples were lysed from three 3.2-mm blood punches by adding 68 μL lysis buffer and 11 μL of proteinase S followed by incubation at 56 °C for 15 min with 900-rpm shaking (ThermoMixer C, Eppendorf, Hamburg, Germany). Ammonium bisulfite (180 μL) and tetrahydrofurfuryl alcohol (TFHA; 60 μL) were added followed by incubation for 45 min at 80 °C, after which binding buffer (580 μL) and isopropanol (380 μL) were added. Punches were removed by transferring the mixture into a fresh 2-mL tube, and magnetic beads (MagBind Particles HDQ) were added for DNA binding. After two extensive washing steps and a drying step at 65 °C for 10–15 min without shaking, 40 μL of elution buffer was added. The samples were incubated at 65 °C for 7–10 min at 1400 rpm, after which the eluate was transferred into fresh 0.2-mL tubes. Converted DNA was stored at -20 °C. For qPCR, 1.5 μL of the DNA was pipetted into a 384-well plate in triplicate, followed by 3.5 μL of the cell-type specific and glyceraldehyde-3-phosphate dehydrogenase (GAPDH)-specific primer/probe master-mix. Two positive controls, a negative control, and a reference sample were prepared for each run in parallel. The plate was sealed and analyzed using the QuantStudio 6 Flex qPCR system (Thermo Fisher, Waltham, MA, USA). Relative (epi) CD3+, CD4+, CD8+, Treg,  and Th17 T cell counts (% of demethylated gene copies of total GAPDH demethylated copies) were calculated as previously described [2].

### 4.3. Data Management and Statistical Analysis

Descriptive statistics were used to summarize the distribution of relative and absolute (epi) lymphocyte counts. Since data did not have a normal distribution, Mann–Whitney U tests were used. For correlation analysis, Pearson r correlation tests were used, while unpaired t-tests were used for group comparison. *p*-values < 0.05 were considered statistically significant. All *p*-values are two-sided. Statistical analysis was carried out with SPSS version 25.0 for Windows (SPSS, Inc., Chicago, IL, USA).

## 5. Conclusions

Epigenetic immune cell counting on DBS is a promising technique for retrospective and prospective immunological research on newborns at a NICU. Our results showed that relative and absolute Treg cell counts were not decreased in NBS cards from preterm neonates who (ultimately) developed a NEC. Even though no evidence was found for pre-existent low Treg numbers and the occurrence of NEC in preterm newborns, we successfully demonstrated the principle of epigenetic immune cell counting for lymphocyte subsets in neonates, both at single or multiple timepoints.

## Data Availability

Data is not publicly accessible, but will be provided upon request.
